# Environmental Risk Assessment of Low Back Pain in ICU Nurses: An Instrument Development Study

**DOI:** 10.1155/2023/3649293

**Published:** 2023-04-19

**Authors:** Lihui Zhang, Yangyang Liu, Su'e Yuan

**Affiliations:** ^1^Teaching and Research Section of Clinical Nursing, Xiangya Hospital of Central South University, Changsha, China; ^2^National Clinical Research Center for Geriatric Disorders, Xiangya Hospital of Central South University, Changsha, China; ^3^Department of Infectious Diseases, Xiangya Hospital, Central South University, Changsha, Hunan, China; ^4^Xiangya Nursing School, Central South University, Changsha, Hunan, China

## Abstract

**Aim:**

To develop a valid, reliable assessment tool to measure risk factors associated to low back pain (LBP) in intensive care unit (ICU) nurses.

**Background:**

LBP is defined as the pain extending from the 12th rib to the iliac crest and often coexists with buttock pain. Nursing has been identified among the top professions at risk of LBP. A mean of 70% prevalence per year in ICU nurses was reported, exceeding those employed in heavy industry. Environmental factors in workplace were also most important risks related to LBP in this population except factors including individual, physical, psychosocial, and lifestyle. However, there is lack of tools to assess environmental risk related to LBP for nurse managers currently.

**Methods:**

Focus group interviews, field research, and panel discussion were used to develop item pool. Two-round expert reviews and preinvestigation were carried out to form initial scale named Environmental Risk Assessing Instrument-Occupational Low Back Pain in Nurses (ERAI-N). A cross-sectional survey with 188 ICU participants in Hunan Province in China was implemented to collect data. Cronbach's alpha, split-half reliability, and test-retest reliability were used to test ERAI-N's reliability. Expert review was performed to test ERAI-N's content validity, and confirmatory factor analysis (CFA) was performed to assess its construct validity, being carried out in IBM SPSS Amos 26 Graphics.

**Results:**

Final version of ERAI-N scale had five dimensions with 18 items that were space, equipment, belief, guideline, and safe culture. ERAI-N scale's score of Cronbach's alpha, Guttman split-half, and intraclass correlation coefficient (ICC) was 0.958, 0.927, and 0.994, respectively. Item-level content validity scores ranged from 0.89 to 1.0, and scale-level content validity was 0.983. Standardized factor loadings ranged from 0.567 to 0.974. Model adjusted fit statistics were as follows: the chi-square statistic and degrees of freedom (*χ*^2^/d*f*) = 3.943, root mean square error of approximation (RMSEA) = 0.071, incremental fit index (IFI) = 0.905, comparative fit index (CFI) = 0.904, parsimony normed fit index (PNFI) = 0.641, and parsimonious comparative fit index (PCFI) = 0.661.

**Conclusions:**

ERAI-N scale had moderate reliability, content validity, and construct validity. *Implications for Nursing Management.* Designers may use ERAI-N scale to plan the interior layout when design a new ICU. Nurse managers might utilize this instrument as a managing tool to assess whether there is environmental risk factors related to LBP in ICU.

## 1. Introduction

Low back pain (LBP) is defined as the pain extending from the 12th rib to the iliac crest and often coexists with buttock pain [1]. LBP is one of the most common public health problems, especially in middle-aged to older women, with the prevalence [2] ranging [3] from 40% to 90% [4]. LBP has some other prominent features such as high annual incidence leading to severe functional limitation [5] in addition to high prevalence. A study done in 195 countries found that LBP was the leading cause of worldwide productivity loss as measured in years and the top cause of years lived with disability [6].

Nursing has been identified among the top professions at risk of LBP [7], especially for those on duty in intensive care unit (ICU). A mean of 70% prevalence per year was reported, exceeding those employed in [8] heavy industry [9]. It is also reported that being a nurse is independently related to spinal pain [10]. LBP can induce disrupted or reduced proprioceptive signaling which likely plays a pivotal role in driving long-term changes in the top-down control of the motor system via motor and sensory cortical reorganization [11]. There were lots of publications which revealed that LBP for ICU nurses may lead to work absenteeism, reduction of nursing workforce efficiency [12], and burnout [13] and may incur economic costs of personal or national finance.

In order to reduce the prevalence of LBP among ICU nurses, investigations have been focused on individual, physical, psychosocial, and lifestyle factors which might play an essential role on the [14] occurrence of LBP [15] in recent decades. Based on human factors theory [16], lots of publications revealed that environmental factors in workplace were the most important risk factors for LBP [17, 18] in ICU nurses [8]. Omura et al. [19] proved that the use of a sliding sheet can significantly lower levels of low back subjective fatigue for caregiver in clinic when performing patient repositioning [19]. Alamgir et al. [18] found that healthcare workers preferred to use ceiling lifts because of less physically demanding work [18]. It is also reported that high-level lift availability was half as likely to have work-related LBP [20]. However, less than half (46%) of respondents working in ICU reported that their employer provided lifts [20], and this means limited availability and adoption of lifting equipment have been a persistent problem [21].

On the other hand, spatial requirements for a bed space are also essential in a critical care setting [22]. It is reported that an average of 23.26 m^2^ was needed for a bed-to-bed transfer followed by 22.87 m^2^ for a resuscitation task [23]. Nevertheless, little investigations were made to make sure whether the bed space in ICU meets the requirements. In terms of culture of safe nursing activities for the prevention of LBP in ICU nurses, there is research indicating that it is urgent to make efforts to broadcast safety operations, to formulate nursing procedures for nurses such as manual handling task, and to carry out guidance for safe handling patients, so as to reduce the occurrence of occupational LBP [24] in ICU nurses [25].

In spite of this, there are currently less reports about these strategies and no established research instruments designed to measure them as well. The aim of the current study was to develop and validate the Environmental Risk Assessing Instrument-Occupational Low Back Pain in Nurses (ERAI-N), with the intent of providing a high-quality instrument with clinical practical value that may be utilized by nurse administrators or researchers.

## 2. Methods

### 2.1. Participating Units

**Figure 1:**
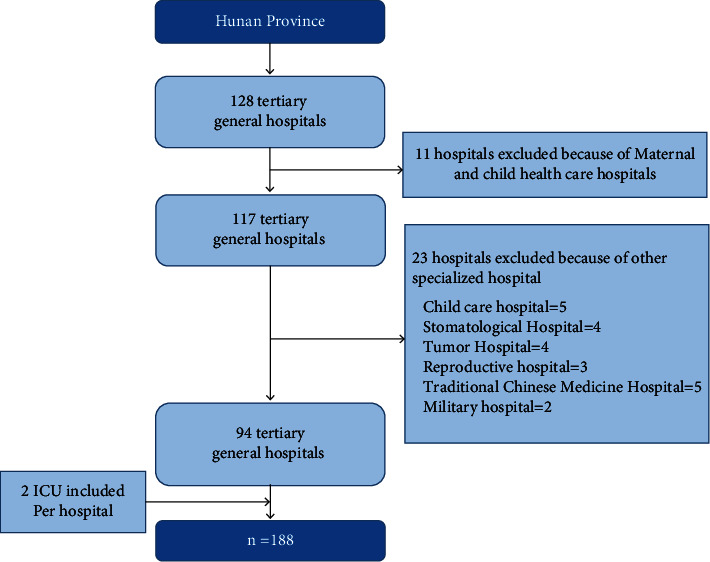
Flowchart of the cluster stratified sampling with random principle.

This was an instrument development and validation study. Sample and data collection was performed in the tertiary general hospitals in Hunan Province in China from March to May 2022, except those specialized hospitals like military hospital, child care hospital, maternal and child health care hospital, stomatological hospital, tumor hospital, reproductive hospital, and traditional Chinese medicine hospital. The final scale used in this investigation has 20 items. The sample size was calculated according to 5 to 10 times of the number of items, 100 to 200 ICUs would be selected for this assessment, and the final sample size was about 110 to 220 ICUs, considering a 10% sample failure rate. There were 2 ICUs being assessed in every tertiary general hospital with random sampling principle. A flowchart of the sampling method is shown in Figure 1.

### 2.2. Procedure

#### 2.2.1. Item Generation

A qualitative study was conducted from January to March 2022. The initial questionnaire was developed through focus group interviews and field research, which were instructed by human factors theory. A convenience sample of 5 head nurses and 3 registered nurses working in a single prestigious general health care center was selected for group interviews. The interview was carried out by 3 of our research team in one conference room in the hospital. Participants were asked to discuss about LBP and environmental risk and safe culture related to the pain. Interviews continued until data saturation was achieved. For further certainty in addition to the focus group interviews, a field research was performed, and descriptive observation and focal observation were used by 2 of our research team in two ICUs. Subsequently, based on the qualitative study and literature review, the preliminary questionnaire containing 27 items was developed.

#### 2.2.2. Content Validity

An expert reviews process based on Delphi [26] was used for this study. The number of experts to be consulted for the Delphi method ranged from 15 to 30, depending on the depth of the research content [27]. Because the instrument is intended to measure factors of working environment and safe culture related to LBP in the context of an inpatient department, 20 researchers in the field of nursing management, ergonomics, or occupational safety were identified as experts, who were from the provinces of Taiwan, Beijing, Hunan, Gansu, Shanghai, Jiangsu, Chongqing, Guangxi and Anhui in China, respectively. All experts were contacted by WeChat and consented to participate in the study.

Two rounds of expert reviews were conducted. Nineteen of these twenty reviewers (95.0%) completed the first round review and 18 of them (94.7%) completed the second round. All of nineteen experts have achieved the top class technique title in the research, and 11 of them had completed postgraduate degree education. For the two-round reviews, the authority coefficient of experts (Cr) was 0.76∼1.00 (0.90 ± 0.08) and 0.75∼1.00 (0.89 ± 0.08), respectively, greater than 0.7; Kendall's W values of the measurement items were 0.223 (*P* < 0.001) and 0.107 (*P* < 0.001); and coefficient of variation (CV) was 0.05∼0.29 (0.11 ± 0.06) and 0.05∼0.22 (0.09 ± 0.03), less than 0.25, indicating that the overall coordination degree of expert scores was at a relatively high level. Experts used Likert 5-score scale to rate each questionnaire item's relevance and its respective concept; a score of 5 means very important, and a score of 1 means not important. The screening criterion was the combination of an item importance score with a mean of ≥4 and CV ≤ 0.25. In the first round, 11 experts put forward suggestions for modification, accounting for more than 57.9%. In the second round, 5 experts put forward suggestions for revision, accounting for 26.3%. After synthesizing expert opinions and group discussions, 6 items were merged, 2 were deleted, 1 item was added, and 2 items were modified. The initial scale with 20 items was obtained.

#### 2.2.3. Preliminary Investigation

To further screen the items of the initial scale that whether their descriptions were suitable for ICU nurse administrators, preinvestigation was performed in 31 ICUs of tertiary medical institutions in Changsha, which were half of nonprofit tertiary hospitals in Changsha. Critical ratio, correlation coefficient, and factor analysis method were used for the screening criteria for project analysis.

### 2.3. Instruments

The final ERAI-N instrument was used as the instrument to collect the data. Participants responded to each item of the scale using a Likert-type scale with five response options: very inconsistent = 1, not very consistent = 2, uncertain = 3, fairly consistent = 4, and very consistent = 5.

### 2.4. Statistical Analysis

#### 2.4.1. Validity Test

After the initial scale was formed, 9 authoritative and highly motivated experts who participated in the first reviews were consulted to test its content validity, evaluating fit degree between the measured content of the scale items and the expected measured content. Experts used the content validity index (CVI) to rate each questionnaire item's relevance and its respective concept. The item CVI (I-CVI) is determined by calculating the proportion of experts rating each item as “quite relevant” or “very relevant.” The I-CVIs were averaged to calculate a scale CVI (S-CVI). I-CVI and S-CVI were used to evaluate item-level and scale-level content validity, respectively.

Construct validity was assessed using confirmatory factor analysis, being carried out in IBM SPSS Amos 26 Graphics. According to the literature, the chi-square statistic and degrees of freedom (*χ*^2^/d*f*) and the root mean square error of approximation (RMSEA) were used as absolute fit indices, comparative fit index (CFI), incremental fit index (IFI), and normed fit index (NFI) were used as incremental fit indices, and parsimony normed fit index (PNFI) and parsimonious comparative fit index (PCFI) were used as parsimonious fit indices [28]. The overall model fit was confirmed to be acceptable when *χ*^2^/d*f* was between 3 and 5 and excellent if it was between 1 and 3. Other acceptable fit criteria were RMSEA < 0.08, CFI > 0.9, IFI > 0.9, NFI > 0.9, PNFI > 0.5, and PCFI > 0.5. Guided by modification indices, residual correlations were specified for several items. Standardized factor loading was used for item-level content validity assessment. Factor loading greater than 0.40 was considered to be adequate. After confirming the model fit, individual standardized parameter estimates of paths (i.e., coefficients values) were assessed for magnitude, statistical significance (*p* ≤ 0.05), and direction.

#### 2.4.2. Reliability Test

Internal consistency reliability was assessed using coefficient alpha and split-half reliability, calculated in SPSS (IBM 26). McDonald's omega reliability coefficient was calculated using SPSSAU (https://spssau.com/). Nunnally and Bernstein [29] suggest that internal consistency reliability values greater than 0.7 are generally sufficient [29]. In this study, test-retest reliability was also performed to assess the instrument's reliability. Forty ICUs were reevaluated 2 weeks after the initial survey. Intraclass correlation coefficients (ICCs) were used to determine test-retest reliability. A series of two-way mixed-effects models with measures of absolute agreement were used. ICCs were determined as <0.40 (poor), 0.40∼0.75 (fair to good), and >0.75 (excellent) [30].

### 2.5. Ethical Considerations

The Medical Research Ethics Committee of Xiangya Hospital of Central South University approved the study protocol (#202109003). Prior to collecting the data, written informed consent was obtained from each participant. The study was conducted in accordance with the Declaration of the World Medical Association and the Helsinki Declaration on the testing of human subjects.

## 3. Results

### 3.1. Characteristics of the Participating Units

**Figure 2:**
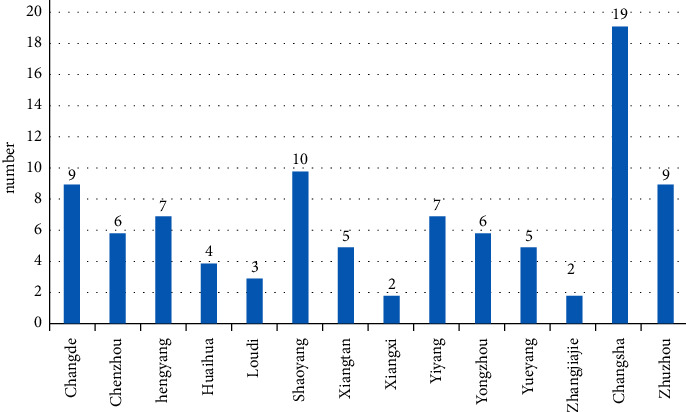
Distribution of the hospital locations.

Of the 94 third-level hospitals surveyed, 19 were in Changsha, accounting for 20.2%, followed by Shaoyang, Changde, and Zhuzhou, with 10 (10.6%), 9 (9.6%), and 9 (9.6%), respectively. The distribution is shown in Figure 2. Fifteen (16.0%) hospitals had <1000 available beds, 50 (53.2%) had 1000–1999 beds, 12 (12.8%) had 2000–2999 beds, and 17 (18.1%) had ≥3000 beds. 56.4% of ICUs were available with 10–19 beds, and 28.7% of ICUs were available with 20 and more beds. In 57.4% of ICUs, half or more of the nurses complained of low back pain due to nursing operations. In 27.7% of ICUs, half or more of the nurses suffered acute low back muscle injuries caused by nursing operations (Table 1).

### 3.2. The Final ERAI-N Instrument

Our hypothetical model, the ERAI-N, theorized that five distinct mechanisms in hospital units contribute to LBP in nurses. These mechanisms are spatial requirement, equipment, belief, guideline, and atmosphere of safe culture. An initial set of items was developed based on human factors theory and was refined through focus group interviews and field research. The final ERAI-N instrument comprises 18 items, with each construct measured by a minimum of 3 and a maximum of 5 items. The total scale score was 18-90. The maximum value in this survey was 90, and the minimum value was 21, with a mean value of 54.7 ± 16.3.

#### 3.2.1. Reliability

*(1) Cronbach's Alpha Score*. Cronbach's alpha scores ranged from 0.793 to 0.982 for the instrument's five factors. Guttman split-half scores ranged from 0.708 to 0.928. McDonald's omega reliability coefficient ranged from 0.866 to 0.986., The overall scale was 0.963 (Table 2). These scores all exceed the 0.7 standard proposed by Nunnally [29].

*(2) Test-Retest Reliability*. In this study, ICC was further analyzed to comprehensively examine the reliability level of the scale, ICC scores ranged from 0.616 to 0.924, and the scale's ICC was 0.994 (Table 3).

#### 3.2.2. Validity

*(1) Content Validity*. In this study, 9 authoritative and highly motivated experts were consulted for the evaluation opinions on the correlation of scale items. The results show that item-level CVI ranged from 0.89 to 1.0, greater than 0.78 [31]. A total of 17 items in the scale were unanimously rated as “relevant” by all experts (3 or 4 points), the scale-level unanimity CVI was equal to 0.85, greater than 0.8 [32], and scale-level average CVI was 0.983, greater than 0.9 [32], which mean the content validity of both item level and scale level was good.

**Figure 3:**
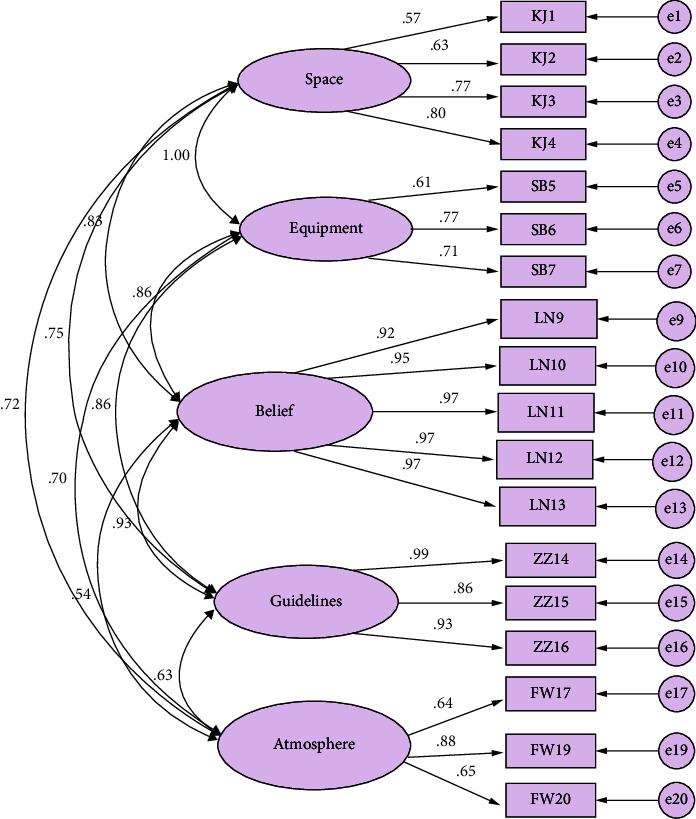
CFA model diagram of Environmental Risk Assessing Instrument-Occupational Low Back Pain in Nurses (ERAI-N).

*(2) Construct Validity*. Construct validity was assessed by confirmatory factor analysis. The maximum likelihood method was used to estimate the factor loadings. In the initial model, *χ*^2^/d*f* was approximately 5, and neither IFI nor CFI was equal to or greater than 0.9. After several times adjusting of model and group discussion, 2 items were deleted. The final model fit indices showed acceptable model fit (Table 4). All parameters in the model were significant at *p* < 0.001 (Figure 3).

The five-factor hypothesized adjusted model resulted in the following goodness-of-fit indices. The standardized factor loadings ranged from 0.567 to 0.974 and exceeded the recommended 0.40 threshold (Table 5).

#### 3.2.3. Minimum Detectable Change

**Figure 4:**
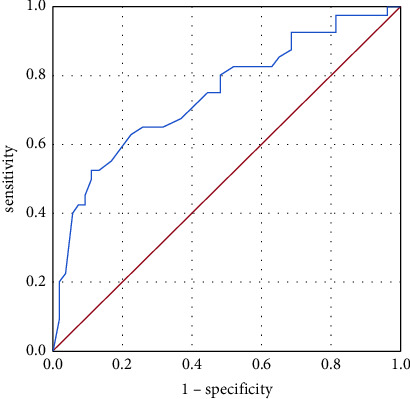
ROC curve.

Nurses' complaints of low back pain due to nursing operations were negatively correlated with scale scores, with a Spearman correlation coefficient of −0.434. Using receiver operating characteristic (ROC) curve analysis, ICUs in which ≥50% of nurses had complained of low back pain due to nursing operations were considered high risk. The AUC was 0.745, *p* = 0.001, the maximum Yordon index was 0.414, and the cutoff value was 62.5 (Figure 4).

## 4. Discussion

Environmental factors in the work place have been found to be an important risk related to LBP in ICU nurses [8, 33]. HealthWISE published by the World Health Organization and International Labour Organization indicated top ten ergonomic principles including working in neutral positions, keeping everything within easy reach, and maintaining a comfortable environment [34]. However, these were instructive principles, and a tool with the capacity of accurately identifying risks in clinic would be more popular than those theoretical principles for clinical administrators. Therefore, we sought to develop such a simple instrument to help clinical administrators complete the identification of environmental risk factors related to LBP in nurses.

### 4.1. Scale Content Analysis

ERAI-N scale had five dimensions with 18 items that were space, equipment, belief, guideline, and safe culture. Jafari et al. [35] developed a scale for predicting LBP occurrence among nurses, including three dimensions with 40 items, which were occupational, psychosocial, and individual [35]. Kazemi [36] developed an instrument to assess occupational low back pain prevention behaviours among nurses, including six aspects that are knowledge, attitude, behaviour, self-efficacy, reinforcing factors, and enabling factors. But the knowledge subscale was with low reliability [36]. However, previous scales related to the low back pain risk assessment in nurses have focused less on the direct and controllable factors that produce these risk behaviours. In addition to safe working procedures [37] and improving nurses' work posture, a safe working environment is also essential. A study showed that providing ceiling lifts was associated with reduced LBP in nurses [21]. Therefore, the instrument is intended to measure factors of working environment and safe culture related to LBP in the context of an inpatient department.

### 4.2. The Results from the Expert Consultation Are Reliable

The experts participating in this review were involved in the fields of nursing management, ergonomics, or occupational safety. The authority coefficient of experts for two reviews was higher than the standard, and it indicated that the experts in this study were highly authoritative. More than half experts put forward suggestions for modification and participated in the consultation for the evaluation of model fit degree, and they fully expressed their concern and support for this study. Furthermore, CV scores were less than 0.25 in two-round reviews indicating that the overall coordination degree of expert scores was at a relatively high level.

### 4.3. ERAI-N Had Moderate Reliability

In terms of the internal reliability, this scale only had a moderate internal consistency.

Reliability analysis is to test the reliability of measuring tools, which is an index reflecting the consistency degree of the result measured from the tool. In this study, coefficient alpha, split-half reliability, and test-retest reliability were used to test ERAI-N's reliability. Coefficient alpha reliability coefficient is the most commonly used reliability coefficient, and it is often used to test scale's intrinsic consistency. The method of split-half reliability means to divide the survey item into two halves and then calculate the correlation coefficient of the scores of the two halves, and it is also used to test scale's intrinsic consistency. For the data analyzed in this study, both coefficient alpha reliability and split-half reliability surpassed generally accepted standards, and this means ERAI-N had moderate reliability. In order to test the results' stability, 40 ICUs were reevaluated 2 weeks later. The scale's ICC was 0.994, greater than the standard of 0.75, indicating that the stability of ERAI-N was excellent [36].

### 4.4. ERAI-N Had Moderate Validity

In this study, in order to ensure the accuracy of phrasing and the importance of entry item, two-round expert consultation was carried out. In the consultation letter, open information collecting columns of “Indicators to be added” and “Opinions and suggestions” was set to obtain experts' advice as more as possible. The evaluation from authoritative experts showed that the CV of both item level and scale level exceeded the standard, this indicated that ERAI-N had excellent content validity, and its items had a good correlation with the corresponding dimension and content.

In order to ensure the scientific research, it is necessary to test the suitability of the questionnaire model. The indices of the initial model including absolute fit indices, incremental fit indices, and parsimonious fit indices basically meet the relevant parameter requirements. Based on it, the initial model was modified in this paper so as to pursue better fitting results. The fitness test results of the modified model showed that *χ*^2^/d*f* was not at the range of excellent, but at the range of fair to good. Meanwhile, CFI, IFI, PNFI, and PCFI reached up to the level of excellent. These findings indicated adequate internal consistency of the items within each factor and a close alignment with the ERAI-N's theoretical factor structure. Therefore, it can be illustrated that ERAI-N had a moderate goodness of fit.

### 4.5. Implications for Nursing Management

There are several clinical and research implications that follow this study. When a new ICU is built, ERAI-N may help the designer to decide the number of sickbed for an area so that a bed space meets ICU spatial requirement standard. It also can help them to determine the location of special equipment such as invasive ventilators that are frequently operated by the nurse so as to keep operators in a natural posture while operating the equipment's interface. Nurse managers can utilize ERAI-N as a managing tool to assess whether there are environmental risk factors related to LBP in their own unit and then form an improvement report and put forward it to supplementary departments. Researchers may tentatively utilize the tool to assess the status quo of environmental risks leading to LBP in ICU.

### 4.6. Limitations of ERAI-N and Future Directions

ERAI-N was developed and tested on a sample from one province; therefore, there is a need for future research focusing on further evaluating the ERAI-N's reliability and validity. For example, it would be national and cross-cultural study with variation in the ERAI-N structure and distribution. The individual factors related to LBP in ICU nurse population can be summarized and set up a connection with the ERAI-N scale. Otherwise, it is urgent to develop practical intervention strategies according to specific conditions for ICU. Of course, it is important for researchers in the future to focus on the study of putting into effect interventions to reduce or eliminate these risk factors, so as to realize its real value of research and development.

## 5. Conclusion

This study introduces a reliable and valid instrument (ERAI-N) for clinical administrators to measure risk factors of low back pain in ICU nurses. The ERAI-N scale had five dimensions with 18 items that were space, equipment, belief, guideline, and safe culture. It demonstrates good levels of reliability, content validity, and construct validity in the process of initial testing and appears promising. Clinical administrators or researchers may tentatively utilize the tool to assess the status quo of environmental risks leading to LBP in ICU. There is a need for future research focusing on further evaluating the ERAI-N's reliability and validity, developing and putting into effect interventions to reduce or eliminate these risk factors, so as to realize its real value of research and development.

## Figures and Tables

**Table 1:** General information of participating units (*n* = 188).

Characteristics	Number (*n*)	Percentage
Type of ICU you work in		
Comprehensive ICU (central ICU)	90	47.9
Specialty ICUs	98	52.1
Respiratory ICU	40	21.3
Emergency ICU	22	11.7
Neurology ICU	8	4.3
Neurosurgical ICU	16	8.5
Cardiac surgical ICU	2	1.1
PICU	4	2.1
Cardiovascular ICU	6	3.2
Number of beds available in your ICU		
<10	28	14.9
10–19	106	56.4
≥20	54	28.7
Have any nurses from your ICU complained of low back pain as a result of nursing operations?		
75–100%	48	25.5
50–74%	60	31.9
26–49%	68	36.2
1–25%	8	4.3
0%	4	2.1
Have nurses from your ICU suffered acute low back muscle injuries as a result of nursing operations?		
75–100%	24	12.8
50–74%	28	14.9
26–49%	82	43.6
1–25%	36	19.1
0%	18	9.6

**Table 2:** Coefficient reliability estimates.

Scale	Coefficient alpha	Split-half reliability	McDonald's omega
Space	0.793	0.708	0.866
Equipment	0.808	0.759	0.875
Belief	0.982	0.938	0.986
Guideline	0.946	0.855	0.965
Atmosphere	0.823	0.806	0.884
The overall scale	0.958	0.927	0.963

**Table 3:** Test-retest reliability.

Dimensionality	The first	The second	ICC
Space	13.55 ± 3.43	13.3 ± 3.96	0.819^*∗∗*^
Equipment	13.60 ± 3.49	13.03 ± 3.93	0.772^*∗∗*^
Belief	14.33 ± 5.82	12.98 ± 5.98	0.924^*∗∗*^
Guideline	8.83 ± 3.36	8.10 ± 3.52	0.834^*∗∗*^
Atmosphere of safe culture	15.43 ± 2.92	14.80 ± 3.07	0.616^*∗∗*^
The overall scale	65.73 ± 16.76	62.20 ± 18.50	0.994^*∗∗*^

ICC, intraclass correlation coefficient. ^*∗∗*^*p* < 0.001.

**Table 4:** Modified model fit test.

Index classification	Indices	Evaluation criterion	Fitted value	Meet the standard
Absolute fit indices	*χ* ^2^/d*f*	<3 good 3∼5 (fail to good)	3.943	Yes
	RMSEA	<0.08	0.071	Yes

Incremental fit indices	IFI	>0.9	0.905	Yes
	CFI	>0.9	0.904	Yes

Parsimonious fit indices	PNFI	>0.5	0.641	Yes
	PCFI	>0.5	0.661	Yes

*χ* ^2^/d*f*, the chi-square statistic and degrees of freedom; RMSEA, root mean square error of approximation; IFI, incremental fit index; CFI, comparative fit index; PNFI, parsimony normed fit index; PCFI, parsimonious comparative fit index.

**Table 5:** Scale items and standardized factor loadings.

Scale items	Standardized factor loadings
Space	
Spatial requirements for a bed space meets the standard of 15 to 18 square meters	0.567
The height of the sickbed is adjustable	0.630
The height of the work surfaces (desks, trolleys, and shelves) is adjustable for nurses working with the natural posture	0.771
The height of the seat and the height of the lumbar support pillow are adjustable, and the structure of the lumbar support pillow is elastic and rigid enough, so it is comfortable and stable	0.798
Equipment	
Auxiliary equipment such as slip and bed easy is available at any time	0.610
Patient lifting system is available at any time	0.772
The height of the interface of common devices is suitable. Nurses do not need to bend or bend excessively when operating the interface (such as monitoring devices and ventilators).	0.709
Belief	
An organizational policy system of working safely has been established	0.917
Promote the concept of “safe patient handling, no manual lift” in the work place	0.952
Training nurses about the knowledge of ergonomics related to the prevention of lumbar and back musculoskeletal injury	0.974
Training nurses on the skill of biomechanics of lumbar spine related to the prevention of lumbar and back musculoskeletal injury	0.973
Evaluate nurses' knowledge and skills in the prevention of lumbar and back musculoskeletal injury	0.965
Guideline	
There were safe work procedures for nurses to prevent low back musculoskeletal injury	0.985
There was risk assessment checklist of safe patient handling	0.856
There were emergency plans to deal with lumbar and dorsal musculoskeletal injuries for nursing staff	0.929
Atmosphere of safe culture	
Nurses have the vision of “I want to work safely”	0.636
Nurses have the initiative to carry out the risk assessment of safe patient handling	0.877
There was a culture of safety named “team work, safe patient handling” in the work environment	0.646

## Data Availability

Data are available on request from the authors.
